# Biomarkers and Proteomics in Sarcomeric Hypertrophic Cardiomyopathy in the Young—FGF-21 Highly Associated with Overt Disease

**DOI:** 10.3390/jcdd11040105

**Published:** 2024-03-29

**Authors:** Anna Wålinder Österberg, Ingegerd Östman-Smith, Henrik Green, Cecilia Gunnarsson, Mats Fredrikson, Petru Liuba, Eva Fernlund

**Affiliations:** 1 Crown Princess Victoria Children’s Hospital, Linköping University Hospital and Division of Pediatrics, Department of Biomedical and Clinical Sciences, Linköping University, SE-58183 Linköping, Sweden; anna.walinder.osterberg@regionostergotland.se; 2 Department of Paediatrics, Institute of Clinical Sciences, Sahlgrenska Academy, University of Gothenburg, SE-41680 Göteborg, Sweden; ingegerd.ostman-smith@pediat.gu.se; 3 Department of Forensic Genetics and Forensic Toxicology, National Board of Forensic Medicine, SE-58185 Linköping, Sweden; 4 Department of Clinical Genetics, Department of Biomedical and Clinical Sciences, Centre for Rare Diseases in Southeast Region of Sweden, Linköping University, SE-58183 Linköping, Sweden; cecilia.gunnarsson@regionostergotland.se; 5 Department of Clinical and Experimental Medicine, Linköping University, SE-58183 Linköping, Sweden; 6 Paediatric Heart Centre, Skåne University Hospital and Department of Clinical Sciences, Lund University, SE-22185 Lund, Sweden; petru.liuba@med.lu.se

**Keywords:** familial hypertrophic cardiomyopathy, genotype, phenotype, biomarkers, proteomics

## Abstract

**Background:** Any difference in biomarkers between genotype-positive individuals with overt hypertrophic cardiomyopathy (HCM), and genotype-positive but phenotype-negative individuals (G+P-) in HCM-associated pathways might shed light on pathophysiological mechanisms. We studied this in young HCM patients. **Methods:** 29 HCM patients, 17 G+P--individuals, and age- and sex-matched controls were prospectively included. We analyzed 184 cardiovascular disease-associated proteins by two proximity extension assays, categorized into biological pathways, and analyzed with multivariate logistic regression analysis. Significant proteins were dichotomized into groups above/below median concentration in control group. **Results:** Dichotomized values of significant proteins showed high odds ratio (OR) in overt HCMphenotype for Fibroblast growth factor-21 (FGF-21) 10 (*p* = 0.001), P-selectin glycoprotein ligand-1 (PSGL-1) OR 8.6 (*p* = 0.005), and Galectin-9 (Gal-9) OR 5.91 (*p* = 0.004). For G+P-, however, angiopoietin-1 receptor (TIE2) was notably raised, OR 65.5 (*p* = 0.004), whereas metalloproteinase inhibitor 4 (TIMP4) involved in proteolysis, in contrast, had reduced OR 0.06 (*p* = 0.013). **Conclusions:** This study is one of the first in young HCM patients and G+P- individuals. We found significantly increased OR for HCM in FGF-21 involved in RAS-MAPK pathway, associated with cardiomyocyte hypertrophy. Upregulation of FGF-21 indicates involvement of the RAS-MAPK pathway in HCM regardless of genetic background, which is a novel finding.

## 1. Introduction

Hypertrophic cardiomyopathy (HCM) is the most common inherited cardiac disease, affecting about 1 of 500 adults, but the disease is less commonly expressed in the pediatric age-range [1,2,3]. The genetic cause is known in about 60% of the cases where the majority have genetic variants in genes encoding for proteins essential to normal myocardial contraction. For familial HCM not associated with any syndrome, the two most affected genetic variants are in the thick myofilament of the sarcomere, MYH7, and MYBPC3, followed by TNNT2 and TNNI3 found in the thin myofilament [4]. In addition, the z-disc of the sarcomere can be affected but this is uncommon, for example in TTN and TCAP [4]. For HCM associated with RASopathy syndrome the most common genetic variant is PTPN11, followed by RAF1 and RIT1 [4,5]. The inheritance is most often autosomal dominant [4]. HCM is the leading cause of sudden cardiac death (SCD) in young individuals, where SCD can be the first sign of the disease [2].

Over the years, several studies have investigated the pathophysiology in HCM with the aim of identifying underlying molecular pathways to increase the understanding of disease progression [6,7,8,9,10]. With better understanding of underlying biological pathways, possible future medicines can be designed to interact at the molecular level. Earlier studies have shown associations between disease progression and high sensitivity Troponin I and NT-proBNP [11,12].

Recently, a large study of plasma biomarkers revealed a novel pathway with an upregulation of the rat sarcoma–mitogen-activated protein kinase (RAS-MAPK) pathway in adult HCM patients compared to controls with left ventricular hypertrophy (LVH) due to hypertension and also confirmed upregulation in pathways involved in inflammation and fibrosis (transforming growth factor beta (TGF-ß) [6]. These biomarkers were in a separate study associated with higher risk of adverse outcome [7]. One proteomic study has identified plasma biomarkers related to hypertrophy and fibrosis (thrombospondin 1, Ras suppressor protein 1, aldolase A, glutathione S-transferase omega 1) as well as inflammation (complement 3) that can discriminate between individuals with clinical diagnosis of HCM and healthy controls, when combined in a proteomics prediction score [13]. There are also studies of protein expression in myocardial samples showing dysregulation of metabolic (creatine kinase, aldolase A) and structural proteins (α-actin, desmin, FHL1) [8]. The field of biomarker profiling in HCM has evolved over recent years, but pediatric and young individuals are still poorly investigated, with only a few available studies [9,14].

The aim of the current study was to determine the association between biomarkers in the recently identified novel pathway affected in HCM, the RAS-MAPK pathway, as well as previously described biological pathways, both in young HCM patients and in those genotype-positive but phenotype-negative individuals at risk of developing the disease.

## 2. Materials and Methods

*Ethical approval* The study was approved by the Regional Ethical Review Board in Lund, Sweden, H15 2009/616, 2011/668 and 2017/522. An informed consent was obtained from all participants or their legal guardians (for participants < 18 years of age). The study conforms to the ethical guidelines of the 1975 Declaration of Helsinki.

*Study design and study participants* This prospective study includes pediatric and young adult patients with hypertrophic cardiomyopathy, genotype-positive but phenotype-negative (G+P-) first-degree relatives at risk of developing the disease and healthy volunteers. The study cohort was consecutively recruited at the Paediatric Heart Centre in Lund, Sweden between 2009–2018, as well as healthy volunteers. Some of the participants have previously been described in a study of biomarkers in HCM in the young [9]. Echocardiography was recorded on the same day as the blood-sample collection.

The study participants were divided into three groups by using echocardiography for measurements of interventricular septum (IVS) and posterior wall (PW) thickness: HCM patients were defined as individuals fulfilling the diagnostic criteria of HCM according to guidelines [3], using Detroit z-score > 2 standard deviations (SD) [15] for individuals <18 years. G+P- individuals were defined as individuals carrying the familial pathogenic variant, but with normal echocardiographic findings. Healthy volunteers matched by age and sex served as controls and had no family history of cardiac disease and a normal echocardiography examination.

Exclusion criteria were HCM patients with RASopathies, secondary HCM due to congenital heart disease, syndromic etiologies, mitochondrial, neuromuscular, or metabolic disorders, or hypertension. HCM patients with left ventricular outflow obstruction (peak gradient > 30 mmHg) and individuals at hereditary risk of HCM without known genetic status were also excluded.

*Transthoracic echocardiography* All transthoracic echocardiography examinations were evaluated by a senior pediatric cardiologist (E.F.) blinded for the results of the biomarkers to verify uniform evaluation. Measurements of IVS and PW were performed and used for inclusion in respective group where HCM was defined as described above [3].

*Genetic testing* Genetic testing using the approved clinical genetic panel for cardiomyopathy at the time of recruitment was performed in the proband (HCM patient), or as pre-symptomatic screening of the identified disease-causing mutation in the first-degree family members. Gene-negative HCM patients underwent further testing using the updated NGS panel in clinical exome, as described previously [16], using clinical exome libraries and prepared using TruSight One Expanded sequencing panel (Illumina) which contains 6794 genes with known clinical significance and sequenced using a NextSeq 500 (Illumina). Variants were assessed according to American College of Medical Genetics and Genomics (ACMG) guidelines [17]. The HCM patients were recruited from 23 unrelated families, and G+P- individuals were recruited from 10 unrelated families. First-degree relatives were tested only for the familial genetic variant according to guidelines. Healthy volunteers did not have any heredity for HCM and hence did not undergo genetic testing.

*Biomarkers* Blood samples were stored as EDTA samples at minus 80 degrees Celsius and sent on dry ice to Olink Bioscience, Uppsala, Sweden. Plasma analyses of 184 cardiovascular disease-associated proteins were performed by two proximity extension assays (Proseek Multiplex CVD-2 and CVD-3, Olink Bioscience, Uppsala, Sweden, Supplementary Table S1 (http://www.olink.com/content/uploads/2021/09/olink-white-paper-pea-a-high-multiplex-immunoassay-technology-with-qpcr-or-ngs-readout-v1.0.pdf (accessed on 26 May 2020)). The biomarkers were log-transformed due to skewed distributions and are presented as normalized protein expression (NPX) values i.e., Olink Proteomics’ arbitrary unit on a log2 scale. Values below limit of detection (LOD) have a lower precise accuracy but are reported and used instead of imputed values such as LOD or 50% of LOD. A quality control of assay performance and quality of individual samples was performed at Olink by adding four internal controls to each sample plate (CVD-2 and CVD-3). Samples not fulfilling the requirements were excluded. Conventional clinical biomarkers were also analyzed including CRP, creatinine, NT-pro-BNP, Troponin T, ALAT, CK MB.

Proteins were categorized into biological pathways according to information at Olink and widely used bioinformatic databases, including Gene Ontology, Uniprot, Human Protein Atlas and DisGeNET. Previously described biological pathways were studied in this study: RAS-MAPK, cell adhesion, response to hypoxia, chemotaxis, immune response, angiogenesis, catabolic process, proteolysis.

*Statistical methods* All statistics were calculated using IBM SPSS Statistics Version27. Continuous variables including age, maximal wall thickness of left ventricle (MWLV) or z-score for MWLV, height, weight and biomarker results were expressed as mean +/− standard deviation (if normally distributed) or median and interquartile range (if not normally distributed). For comparisons between groups, two-sided *t*-test or Mann–Whitney u-test were used as appropriate for type of distribution. A result with *p* < 0.05 was considered statistically significant. To investigate any independent biomarker/biomarkers in the different pathophysiologic pathways, explorative analysis was conducted using multivariate logistic regression analysis with stepwise, forward entry, adjusted for age and sex, comparing the HCM group with their matched controls, as well as the G+P- group with their matched controls. To visualize differences in significant protein concentration between the study groups a binary logistic regression model with stepwise, forward entry, adjusted for age and sex, was conducted using dichotomized values of significant proteins from each pathophysiological pathway. The significant proteins were dichotomized into two groups, above or below median protein concentration in the combined control groups (matched controls of HCM and G+P- together). We used the same methodology in all pathway analysis. Each multivariable and binary logistic regression model generated exclusive conditions for each pathway analysis and hence adjustment for multiple comparisons was not appropriate.

## 3. Results

### 3.1. Study Participants

The study cohort originally consisted of 96 individuals: 30 HCM patients, 18 G+P- individuals and 48 controls matched by age and gender. Two samples (one HCM patient and one G+P- individual) did not pass the quality control and hence were excluded together with their matched controls. After exclusion, the study cohort consisted of 92 individuals: 29 HCM patients with 29 controls matched by age and sex and 17 G+P- individuals with 17 controls matched by age and sex. The two control groups for HCM and G+P- are referred to as their controls. Demographics and echocardiographic details of the study groups are presented in Table 1. The gene panels tested were positive in 22 of 29 HCM cases using standard panels (76%). Gene-negative HCM cases underwent extended genetic analysis, which detected pathological genetic variants in two of them. In total, 24 of 29 individuals (83%) were found to have causative genetic variants for HCM. The genetic distribution in the groups of HCM patients and G+P- individuals are presented in Table 2 and minor allele frequencies are presented in Supplementary Table S2. Inclusion of age and sex in the regression models did not affect the results.

### 3.2. Transthoracic Echocardiography

Echocardiographic findings are presented in Table 1. IVS and PW dimensions are presented as mm, Detroit z-score, and Boston z-score.

### 3.3. Biomarkers and Pathophysiological Pathways in the Study Groups

The biomarkers in the current study were grouped into biological pathways according to functional entities at Olink Bioscience, Uppsala, Sweden (see Methods). The clinical routine biomarkers of the study participants are shown in Table 1 and in Supplementary Table S3.

#### 3.3.1. RAS-MAPK Pathway

The RAS-MAPK pathway was analyzed by studying 40 proteins involved in the pathway. In the model analyzing differences between HCMs and their controls, one protein was significantly increased: Fibroblast growth factor 21 (FGF-21), Table 3, (including results that remained significantly different after dichotomization) and two proteins were significantly decreased: interleukin-6 receptor subunit alpha (IL-6RA), Table 3 and interleukin-2 receptor subunit alpha (IL2-RA), Supplementary Table S4 (including results that did not remain significantly different after dichotomization). FGF-21 had a high odds ratio (OR) 53.09 (*p* = 0.023), i.e., every increase of one unit in protein concentration increased the associated risk for overt HCM phenotype while IL2-RA and IL-6RA had OR below 1 (*p* = 0.043 and *p* = 0.027), i.e., decreased associated risk of HCM phenotype with increasing protein values. When including the dichotomized variables in the binary logistic regression model, FGF-21 and IL-6RA remained significantly different between the groups with OR 10.0 (*p* = 0.001) and OR 0.26 (*p* = 0.041) respectively for HCM, Table 3.

In the model analyzing differences between G+P- and their controls none of the proteins in this pathway reached statistical significance.

#### 3.3.2. Cell Adhesion

We analyzed 62 proteins involved in cell adhesion. In the model analyzing differences between HCM and their controls three proteins were significantly increased: P-selectin glycoprotein ligand-1 (PSGL-1), Table 3, interleukin-1 receptor antagonist protein (IL-1ra) and intercellular adhesion molecule 2 (ICAM-2), Supplementary Table S4. Two proteins were significantly decreased: epidermal growth factor receptor (EGFR), Table 3, and interleukin-6 (IL-6), Supplementary Table S4. When analyzing the dichotomized variables, PSGL-1 (OR 8.6; *p* = 0.005), and EGFR (OR 0.1; *p* = 0.013), remained significantly different between overt HCM and controls, Table 3.

In the model analyzing differences between G+P- and their controls none of the proteins reached statistical significance.

#### 3.3.3. Response to Hypoxia

We analyzed 17 proteins involved in the response to hypoxia. In the model analyzing differences between HCM and their controls, one protein was significantly increased with a high OR for HCM: pro-adrenomedullin (ADM) (*p* = 0.008) and one protein was significantly decreased with an OR < 1 for HCM: matrix metalloproteinase-2 (MMP-2) (*p* = 0.008). Neither protein remained significant when analyzing the dichotomized variables, Supplementary Table S4.

In the model analyzing differences between G+P- and their controls none of the proteins reached statistical significance.

#### 3.3.4. Chemotaxis

We analyzed 17 proteins involved in chemotaxis. In the model analyzing differences between HCM and their controls one protein: interleukin-6 receptor subunit alpha (IL-6RA) was significantly decreased, with an OR < 1 (*p* = 0.022) for HCM but when analyzed as a dichotomized variable in a binary logistic regression model, IL-6RA did not reach statistical significance, Supplementary Table S4.

Similarly, in the model analyzing differences between G+P- and their controls one protein, CD166 antigen (ALCAM), was significantly decreased between the groups, with OR < 1 (*p* = 0.017), but failed to reach statistical significance when analyzed as a dichotomized variable, Supplementary Table S5.

#### 3.3.5. Immune Response

The immune response was studied by analyzing 38 proteins involved in the system. In the model analyzing differences between HCM and their controls two proteins were significantly increased with high OR for HCM: galectin-9 (Gal-9) (*p* = 0.044), Table 3, and polymeric immunoglobulin receptor (PIgR) (*p* = 0.008), Supplementary Table S4. Two proteins were significantly decreased with OR < 1 for HCM: programmed cell death 1 ligand 2 (PD-L2) (*p* = 0.02) and osteoclast-associated immunoglobulin-like receptor (hOSCAR) (*p* = 0.025), Supplementary Table S4. When analyzing the dichotomized significant proteins, Gal-9 remained significantly (*p* = 0.004) increased with OR 5.9 for HCM, Table 3, while PIgR, PD-L2 and hOSCAR did not reach statistical significance, Supplementary Table S4.

In the model analyzing differences between G+P- and their controls one protein: A disintegrin and metalloproteinase with thrombospondin motifs 13 (ADAM-TS13) was significantly increased between the groups, Table 4, and showed a high OR (*p* = 0.007) for G+P. ADAM-TS13 remained significantly (*p* = 0.034) increased with OR 11.2 for G+P-, Table 4, when analyzing the dichotomized variable.

#### 3.3.6. Angiogenesis

In angiogenesis, 28 proteins were included. In the model analyzing differences between HCM and their controls, three proteins were significantly increased with high OR for HCM: decorin (DCN) (*p* = 0.049), plasminogen activator inhibitor 1 (PAI) (*p* = 0.018), and integrin beta-2 (ITGB2) (*p* = 0.026), Supplemenary Table S4. One protein was significantly decreased with OR < 1 for HCM: MMP-2 (*p* = 0.005), Supplemenary Table S4. When analyzing the dichotomized significant proteins, none of the proteins remained significantly different between the groups, Supplemenary Table S4.

In the model analyzing differences between G+P- and their controls one protein was significantly increased with high OR for G+P-: angiopoietin-1 receptor (TIE2) (*p* = 0.048) and one protein was significantly decreased with OR < 1 for G+P-: aminopeptidase N (AP-N) (*p* = 0.019), Table 4. When analyzing the dichotomized variables both TIE2 (*p* = 0.004) and AP-N (*p* = 0.032) remained significantly different between the groups, Table 4.

#### 3.3.7. Proteolysis

We analyzed 34 proteins involved in proteolysis. In the model analyzing differences between HCM and their controls, none of the proteins reached statistical significance.

In the model analyzing differences between G+P- and their controls two proteins were significantly decreased with OR < 1 for G+P-: metalloproteinase inhibitor 4 (TIMP4) (*p* = 0.042) and PAI (*p* = 0.03), Table 4, and one protein was significantly increased with high OR for G+P-: urokinase plasminogen activator surface receptor (U-PAR) (*p* = 0.035), Supplemenary Table S5. When analyzing the dichotomized variables, TIMP4 and PAI remained significantly decreased between the groups with OR 0.06 (*p* = 0.013) and 0.08 (*p* = 0.031) respectively for G+P-, Table 4.

#### 3.3.8. Pathways Analyzed without Significant Results

Proteins involved in inflammatory response (59 proteins) and catabolism (43 proteins) were analyzed. None of the proteins reached statistical significance in the models analyzing differences between HCM and their controls nor in models analyzing differences between G+P- and their controls.

## 4. Discussion

Early studies of biomarkers showed the importance of Troponin T as a marker of myocardial damage [11] and proBNP as a reflection of increased loading conditions [12], now routinely evaluated in cardiac disease [3]. The current study is one of few studies of biomarkers in the pediatric and young HCM population and shows several important findings supporting previous knowledge of the pathogenesis in HCM and some new findings.

Several possible pathophysiological pathways involved in the developing phenotype of HCM have been described over the years [4,6,7,8,9,10]. In this study we aimed to investigate any differences in protein expression, between HCM patients and age- and sex-matched controls as well as between G+P- individuals and their controls.

### 4.1. Proteins Associated with Increased Risk of Disease-Expression

*FGF-21:* HCM is a common associated feature in RASopathies i.e., disorders caused by genetic variants in genes encoding for components in the RAS-MAPK cascade [5]. Shimada et al. showed an upregulation of the RAS-MAPK pathway in HCM patients without RASopathies [19]. Patients with RASopathy-associated HCM were excluded from our study and we still find an upregulation of FGF-21 with an OR for HCM of 10.0 (*p* = 0.001) compared with their controls, using the dichotomized variables. FGF-21 is a protein involved in the RAS-MAPK pathway and contributes to multiple biological processes by its function as a metabolic regulator. Regarding FGF-21, there is conflicting evidence in the literature. On one hand Zhang et al. described a diabetic mouse model with streptozotocin-induced diabetic mice. They treated diabetic and control mice with FGF-21 for 10 days and showed, by TUNEL staining, that FGF-21 prevented diabetes-induced cardiac apoptosis and hypothesized a cardiac protective role of FGF-21 administration [20]. On the other hand, Ferrer-Curriu et al. showed, in cardiac samples from adult hypertensive patients, a correlation between increasing FGF-21 levels expressed in myocardium and degree of cardiomyocyte hypertrophy and increasing degree of interstitial fibrosis [21]. The only study of HCM and FGF-21 is a study by Viola et al. in a mouse model corresponding to the human MYH7 Arg403Gln [22]. They studied the role of the L-type calcium channel (L-Ca-L) in regulating mitochondrial function and found an increase of metabolic activity when activating the L-Ca-L in genetically altered mice, as well as an increase in FGF-21, also known to be a marker of mitochondrial dysfunction. The increase of FGF-21 correlated with the onset of cardiomyopathy [22]. The FGF-21 findings by Viola et al. in a mouse model support the importance of the findings in the present study, with significantly higher levels of FGF-21 in HCM patients compared to healthy controls. Our study is the first study showing this in HCM in humans. In line with this we did not find any differences between G+P- individuals i.e., without onset of cardiomyopathy, and their controls.

*PSGL-1:* The protein PSGL-1 is involved in cell adhesion and was also increased in HCM patients as compared to their controls, an increase that remained after dichotomization with an OR for HCM of 8.6 (*p* = 0.005). PSGL-1 is expressed on different types of leukocytes and plays an important role of the initial steps of inflammation by binding P-selectin, expressed at the surface of activated platelets and endothelium [23] as well as in supporting neutrophil rolling by binding P-selectins [24]. Elevated levels of PSGL-1 in HCM patients are not previously described and is in line with theories of proinflammatory stages in the development of the disease [7,25].

*GAL-9:* A pro-inflammatory stage is also thought to play a role in HCM progression [7,25]. In the current study we found significantly elevated levels of Gal-9 in the HCM group even after dichotomization below or above median concentration, Table 3. Gal-9 is a protein belonging to the galectin family and is included among the matricellular proteins. It is produced by endothelial cells where the production increases after interferon-γ stimulation. Interferon-γ plays an important role in the immune system, regulating the function of lymphocytes and macrophages [26]. Gal-9 has also been shown to be expressed in vascular endothelial and inflammatory cells, such as fibroblasts [27] and acts as an eosinophil chemoattractant promoting eosinophil adhesion to vascular endothelium [28]. Elola et al. suggested involvement of Gal-9 in the process of developing fibrosis by the possible contribution of eosinophil recruitment and infiltration [28]. In the current study, we found an increased odds ratio of 5.9 (*p* = 0.004) for HCM. This has not been shown earlier for HCM. This difference was not seen between G+P- and their controls, supporting the theory of involvement of the immune system and development of fibrosis during the development of overt disease.

### 4.2. Proteins That Are Reduced in Overt HCM

*EGFR* is involved in cell adhesion. There a few studies describing an upregulation of EGFR leading to remodelling of the desmosome by tyrosine phosphorylation of the plakoglobin, changing the interaction with desmoplakin in the desmosome, and you can speculate if this has implications in the pathogenesis of arrhythmogenic right ventricular cardiomyopathy (ARVC) [29]. In HCM, EGFR is not well described and there are a few animal studies recently publicized, describing activation of the EGFR pathway contributing to impaired relaxation in HCM [30]. However, we found changes in the opposite direction in human HCM with an OR for HCM of 0.1 (*p* = 0.013), Table 3.

### 4.3. Proteins That Are Altered in Genotype-Positive Individuals without Developed HCM Phenotype (G+P-)

*ADAM-TS13:* In the current study there was a significantly increased level of ADAM-TS13 between G+P- compared to their controls (OR 11.2, *p* = 0.03), but not when comparing HCM and their controls. ADAM-TS13 is involved in the immune response and belongs to a family of extracellular matrix metalloproteases (ADAM-TS) together with the membrane-bound A disintegrin and metalloproteinase (ADAM). ADAM-TSs are enzymes secreted extracellularly while ADAMs are important mediators of cell signalling, mostly intracellularly. ADAM-TS13 has not been studied in cardiomyopathies previously but several ADAM-TSs (1, 5 and 9) have been reported to be expressed in embryologic development of the heart. ADAM-12 and -17 are thought to be involved the development of hypertrophy with ADAM-12 acting as a mediator of hypertrophy regulated by ADAM-17 [31]. One can speculate if the current result of increased levels of ADAM-TS13 in G+P- is a sign of activity in the myocardium before the evolvement from phenotype-negative to phenotype-positive stature. ADAM-TS13 is also known as von Willebrand factor (vWF)-cleaving protease and is involved in platelets thrombus, preventing uncontrolled blood clotting. ADAM-TSs are inhibited by metalloproteinase inhibitors (TIMPs) [31].

*TIMP4:* In the current study TIMP4 remained significantly lower in G+P- after dichotomization in the proteolytic pathway (OR 0.06, *p* = 0.01) compared to their controls G+P-. One can speculate if a reduced inhibition by TIMP4 might be the explanation for the raised ADAM-TS13 levels, but we have no additional evidence to support that hypothesis. Only a few studies have been performed in individuals at hereditary risk at HCM but none of them include ADAM-TS13 and TIMP4 identified in this study. Fernlund et al. found two significant proteins (ICAM-1 and myostatin) in a prior study [9] compared to controls and compared to HCM, these proteins were not studied in the current study.

*TIE2 and AP-N:* After dichotomization of significantly different proteins involved in angiogenesis none of the proteins remained significantly different between HCM and their controls. However, both proteins remained significantly different after dichotomization in the G+P- individuals compared to their controls, where TIE2 showed increased risk of mutation carriage (OR 66, *p* = 0.004) and AP-N reduced risk (OR 0.08, *p* = 0.03). TIE2 is an important endothelial cell-surface receptor for angiopoietin-1 (ANGPT1) and angiopoietin-2 (ANGPT2) and involved in angiogenesis. ANGPT1 acts as an agonist to TIE2, stimulating vessel stability when binding to the receptor, while ANGPT2 acts as an antagonist leading to vessel permeability, instability, and remodelling [32]. Several studies have described the ANGPT/TIE2-pathway as a possible target for anti-angiogenesis therapy in cancer [33,34] and in inflammatory bowel disease [35]. In the current study we showed increased levels of TIE2 in G+P- individuals compared to their controls. This indicates a possible upregulation of TIE2 in G+P- and you can speculate if this upregulation prevents the evolvement to developed phenotype. TIE2 is also expressed at the surface of macrophages, shown to be activated in inflammation, matrix remodelling and involved in angiogenesis [36]. You can also speculate if there is an imbalance in the ANGPT/TIE2 pathway with a shift from ANGPT1 to ANGPT2 during the disease progression in HCM leading to disturbed angiogenesis. Thus, it is conceivable that increased levels of TIE2 (and possibly also ADAM-TS13) may protect a genotype-positive individual from developing the phenotype.

*AP-N* belongs to the family of aminopeptidase and has been reported to be involved in angiogenesis as the third protease system, where the other two systems are plasminogen activators (PAs) and matrix metalloproteinases (MMPs) [37,38]. Aminopeptidases are proteolytic enzymes involved in protein maturation, regulation, and degradation where AP-N is a membrane-bound zinc-dependent metalloproteinase. AP-N has been shown to be involved in cardiac remodelling after myocardial infarction [37], expressed in endothelial cells on blood vessels undergoing angiogenesis but not expressed in normal vasculature [38] and has also been shown to have dysregulated expression in inflammatory disease [39]. AP-N is also known to induce transcription of MMPs and to be involved in the renin–angiotensin system, where it converts angiotensin III to IV by its proteolytic activity [40]. There are several studies investigating the role of AP-N in angiogenesis, especially in tumour cells where angiogenesis is a prerequisite for tumour growth, and a potential target for drugs [38,41]. In current study we found decreased OR (0.08, *p* = 0.03) for G+P- compared to their controls. You can speculate if this indicates consumption of AP-N in G+P-individuals leading to the thought of unknown mechanisms inhibiting the onset of disease evolvement including inhibiting angiogenesis. To the best of our knowledge any change of AP-N levels in HCM evolvement has not been described earlier and further research could give insights in mechanisms triggering the onset of overt disease in G+P- individuals.

*PAI* was significantly increased in HCM compared to their controls (OR 40.5, *p* = 0.018) but decreased comparing G+P- and their controls (OR 0.005, *p* = 0.03). After dichotomization PAI remained significantly reduced comparing G+P- and their controls (OR 0.08, *p* = 0.03) but did not remain significantly increased for HCM. PAI is a member of the serine protease inhibitor family, inhibiting the plasminogen system, negatively regulates the activation of MMPs and is important in tissue homeostasis and angiogenesis. PAI is well investigated in liver, skin, lung, and kidney and has a profibrotic effect by inhibiting the plasmin–MMP system responsible for the elimination of collagen and fibrin. On the contrary, experimental studies have shown that genetic deficiency in PAI-1 promotes spontaneous cardiac-selective development of fibrosis in aging mice as well as in humans [42]. Flevaris et al. suggested PAI-1 to be a molecular switch by showing a regulatory mechanism of PAI-1 for cardiac transforming growth factor-β (TGF-β) synthesis. Deficiency in PAI-1 resulted in an activation of TGF-β synthesis, enhancing the transcriptional targets involved in extracellular matrix remodelling, matricellular proteins and extra cellular matrix components [42]. There are no studies in HCM patients, but you can speculate if the results by Flevaris et al. is a contributing explanation to the findings in our study showing an increased OR for HCM but a decreased OR for G+P- individuals. Could PAI-1 be upregulated in overt HCM as a response to the extracellular matrix deposition occurring together with cardiac hypertrophy and fibrosis development, whereas G+P- individuals have not yet progressed to that stage?

### 4.4. Implications for the Future

The results of the current study strengthen the importance of future large-scale biochemical studies to improve the understanding of the pathophysiology in disease development in young individuals with HCM. Several of the proteins identified in this study are involved in extracellular matrix turnover, remodelling, and angiogenesis. It would be of interest to study inhibition of activated proteins, such as galectin-9 and TIE2, in animal models. The current study is an explorative hypothesis-generating study, and it would also be of interest to study changes over time in the present cohort to better understand differences in the young and adult HCM patient.

### 4.5. Limitations of the Study

HCM is a rare disease in childhood resulting in a limited number of patients in the current study. Due to the inherent statistical uncertainty caused by a small sample size, this study risks missing small changes between the groups that might have been significant in larger sample sizes. The explorative study design is not the optimal way of investigating biological mechanisms but is an important way of directing further studies.

## 5. Conclusions

Our current biochemical study is one of the first in young HCM patients and G+P- individuals and shows a significantly increased OR for HCM in FGF-21. FGF-21 is part of the RAS-MAPK pathway and seems to be involved in HCM regardless of genetic background, which is a novel finding. We have also shown significant changes in several other proteins important in angiogenesis, protein turnover, cell signalling and in proinflammatory stages. Interestingly, the pattern of protein changes in genotype-positive, phenotype-negative individuals is different from that in overt disease, possibly giving clues to disease-modifying mechanisms. Our findings encourage further large-scale proteomics studies in HCM patients and in G+P- individuals to reinforce the findings of the current study. Future proteomic studies with large HCM and G+P- cohorts with greater statistical power might identify new significant findings—enhancing the understanding of the mechanism of disease progression and thus identifying possible new targets for treatment.

## Figures and Tables

**Table 1:** Clinical characteristics of the study groups (92 individuals) compared between HCM and age- and sex-matched controls (matched controls) and between genotype-positive but phenotype-negative (G+P-) individuals and their age- and sex-matched controls (matched controls).

	HCM (n = 29)	Matched Controls (n = 29)	*p* Value *^/†^	G+P- (n = 17)	Matched Controls (n = 17)	*p* Value *^/†^
Male/Female, n/n (% male)	22/7 (76)	22/7 (76)	N/A	10/8 (56)	9/8 (53)	N/A
Age (years), median [18]	18.0 [8.0]	19.1 [8.0]	0.176 *	13.1 [8.4]	17.0 [7.5]	0.107
Heredity, n	29	N/A	N/A	17	N/A	N/A
mm	16.0 [8.0]	10.0 [2.1]	<0.001 *	8.8 [3.2]	9.2 [1.9]	0.900 *
MWT IVS median [IQR] z-score ^a^	7.6 [9.4]	1.7 [1.5]	<0.001 *	2.1 [1.9]	1.4 [1.4]	0.260 *
z-score ^b^	2.9 [2.8]	0.7 [0.8]	<0.001 *	1.3 [0.9]	0.8 [1.1]	0.053 *
mm	10.0 [3.2]	9.2 [1.3]	0.009 *	8.2 [2.4]	8.1 [1.3]	0.112 *
MWT PW median [IQR] z-score ^a^	2.2 [3.2]	0.8 [1.7]	0.002 *	0.5 [2.0]	0.6 [1.3]	0.450 *
z-score ^b^	1.44 [1.6]	0.7 [0.8]	<0.001 *	0.8 [0.9]	0.9 [0.9]	0.763 *
NT-proBNP ng/L, mean (SD) (<450)	545.1 (881.1)	49.4 (1.88)	**0.004 ^†^**	60.1 (30)	49.5 (1.4)	0.143 ^†^
Troponin T ng/L, mean (SD) (<15)	12.33 (22.4)	5.65 (1.99)	0.115 ^†^	4.6 (2.2)	5.3 (0.8)	0.486 ^†^

z-score ^a^ = Boston 2D z-score; z-score ^b^ = Detroit z-score; *^/†^ Between-group differences were evaluated using Mann–Whitney * Test or Two-Sided *t*-test ^†^; *p* value < 0.05 was considered statistically significant and written in bold; [IQR] = inter-quartile range; (SD) = standard deviation; N.A. = not applicable; MWT = maximal wall thickness by echocardiography; IVS = interventricular septum; PW = posterior wall.

**Table 2:** Genetic variants found in the HCM and genotype-positive, phenotype-negative (G+P-) groups. Genetic testing using the approved clinical genetic panel for cardiomyopathy at the time of recruitment was used as initial screening. Gene-negative HCM patients underwent further testing using the updated next generation sequencing (NGS) panel in clinical exome. HCM patient without identifying a disease-causing genetic variant after the described further testing were described as without identified known pathogenic variant. One HCM patient had two pathogenic variants (in MYH7 and MYBPC3).

	HCM (n = 29)	G+P- (n = 17)	Healthy Volunteers (n = 46)
Genetic spectrum (n)	*MYBPC3* (15)	*MYBPC3* (7)	N/A
	*MYH7* (6)	*MYH7* (7)	
	*PRKAG2* (1)	*TNNT2* (3)	
	*TCAP* (1)		
	*TNNI3* (1)		
	ABCC9 (1)		
	Without identified known pathogenic variant (5)		

*N.A. = not applicable.*

**Table 3:** Descriptive statistic and multivariable logistic regression models, adjusted for age and sex in pathophysiological pathways when comparing HCM (n = 29) with age- and sex-matched controls (matched controls) (n = 29) in each model. Significant proteins in each pathway are presented as increased or decreased OR for HCM. Significant proteins are dichotomized above or below the median value of each protein in the control groups and included in a binary logistic regression model for each pathway, adjusted for age and sex. Proteins remaining statistically significant in the binary model are presented here.

	HCM (n = 29)	Matched Controls (n = 29)	Model with Continuous Protein Values		Model with Dichotomized Protein Values	
	Median (IQR)	Median (IQR)	OR (95% CI)	*p*-Value ^†^	OR (95% CI)	*p*-Value ^†^
**Ras MAPK** (40 proteins) *						
**FGF-21**	5.83 (2.17)	3.58 (1.40)	53.09 (1.72–1639)	**0.023**	10.0 (2.5–39.6)	**0.001**
**IL-6RA**	12.87 (0.74)	13.26 (0.51)	0.005 (“0”–0.55)	**0.027**	0.26 (0.07–0.95)	**0.041**
**Cell adhesion** (62 proteins) *						
**PSGL-1**	6.12 (0.34)	5.80 (0.20)	4.65 × 10^12^ (19.5–1.1 × 10^24^)	**0.029**	8.6 (1.9–39.0)	**0.005**
**EGFR**	3.75 (0.50)	3.91 (0.55)	5.7 × 10^−11^ (“0”–0.03)	**0.022**	0.13 (0.03–0.65)	**0.013**
**Immune response** (38 proteins) *						
**Gal-9**	9.36 (0.44)	9.02 (0.48)	60.6 (1.12–3275)	**0.044**	5.91 (1.8–19.8)	**0.004**

* Number of proteins analyzed and expressed as NPX = normalized protein expression values, Olink Proteomics’ arbitrary unit on log2 scale; inter quartile range (IQR); “0” = <0.001; *p* ^†^ = *p* value < 0.05 was considered statistically significant.

**Table 4:** Descriptive statistic and multivariable logistic regression models, adjusted for age and sex, and separated in pathophysiological pathways when comparing genotype-positive, phenotype-negative (G+P-) (n = 17) with age- and sex-matched controls (matched controls) (n = 17) in each model. Significant proteins in each pathway are presented as increased or decreased OR for G+P-. Significant proteins are dichotomized above or below the median value of each protein in the control groups and included in a binary logistic regression model for each pathway, adjusted for age and sex. Proteins remaining statistically significant in the binary model are presented here.

	G+P- (n = 17)	Matched Controls (n = 17)	Model with Continuous Protein Values		Model with Dichotomized Protein Values	
	Median (IQR)	Median (IQR)	OR (95% CI)	*p*-Value ^†^	OR (95% CI)	*p*-Value ^†^
**Immune response** (38 proteins) *						
**ADAM-TS13**	6.52 (0.32)	6.22 (0.32)	598 (5.8–61607)	**0.007**	11.2 (1.2–105)	**0.034**
**Angiogenesis** (28 proteins) *						
**TIE2**	8.66 (0.43)	8.31 (0.85)	200 (1.1–37978)	**0.048**	65.5 (3.7–1165)	**0.004**
**AP-N**	5.72 (0.54)	6.02 (0.70)	0.0 (“0”–0.28)	**0.019**	0.08 (0.008–0.81)	**0.032**
**Proteolysis** (34 proteins) *						
**TIMP4**	3.59 (0.85)	4.15 (0.60)	1.44 × 10^−8^ (“0”–0.5)	**0.042**	0.056 (0.006–0.54)	**0.013**
**PAI**	3.80 (1.60)	4.58 (1.53)	0.005 (“0”–0.6)	**0.030**	0.084 (0.009–0.80)	**0.031**

* Number of proteins analyzed and expressed as NPX = normalized protein expression values, Olink Proteomics’ arbitrary unit on log2 scale; inter quartile range (IQR); “0” = <0.001; *p* ^†^ = *p* value < 0.05 was considered statistically significant.

## Data Availability

The data presented in this study are available on reasonably request from the corresponding author.

## Supplementary Materials

The following supporting information can be downloaded at: https://www.mdpi.com/article/10.3390/jcdd11040105/s1, Supplementary Table S1: Biomarkers in respectively biological pathway. Supplementary Table S2. Genetic variants with minor allele frequencies for each genetic variant. Supplementary Table S3. Clinical markers compared between HCM and age- and sex-matched controls and between genotype-positive, phenotype-negative (G+P-) individuals and age- and sex-matched controls. Supplementary Table S4. Descriptive statistic and multivariable logistic regression models, adjusted for age and sex in pathophysiological pathways when comparing HCM with age- and sex-matched controls in each model. Significant proteins are dichotomized above or below the median value of each protein in the control groups and included in a binary logistic regression model for each pathway, adjusted for age and sex. Supplementary Table S5. Descriptive statistic and multivariable logistic regression models, adjusted for age and sex, in pathophysiological pathways when comparing phenotype-negative, genotype-positive individuals (G+P-) with age- and sex-matched in each model. Significant proteins are dichotomized above or below the median value of each protein in the control groups and included in a binary logistic regression model, adjusted for age and sex, for each pathway.
