# Supplementary material for: Biomarkers and Proteomics in Sarcomeric Hypertrophic Cardiomyopathy in the Young—FGF-21 Highly Associated with Overt Disease
Source: J Cardiovasc Dev Dis. 2024 Mar 29;11(4):105. doi: 10.3390/jcdd11040105 (PMC11050055; doi:10.3390/jcdd11040105)
Supplement: Supplementary file 1 [file jcdd-11-00105-s001.zip › jcdd-2914809-supplementary.pdf]

Supplementary tables S1–5

Table S1. Biomarkers in respectively biological pathway.

**Biomarkers in Immune response (38 proteins)**	
Tumor necrosis factor receptor superfamily member 13 B (TNFRSF13B)*	Interleukin-1 receptor-like 2(IL1RL2)*
NF-kappa-B essential modulator (NEMO)*	T-cell surface glycoprotein CD4 (CD4)*
Lymphotactin (XCL1)*	Interleukin-6 (IL6)*
Low affinity immunoglobulin gamma Fc region receptor II-b (IgG Fc receptor II-b)*	C-C motif chemokine 3 (CCL3)*
Proto-oncogene tyrosine-protein kinase Src (SRC)*	Interleukin-18 (IL-18)*
Carcinoembryonic antigenrelated cell adhesion molecule 8 (CEACAM8)*	Galectin-9 (Gal-9)*
Heme oxygenase 1 (HO-1)*	Polymeric immunoglobulin receptor (PIgR)*
Interleukin-4 receptor subunit alpha (IL-4RA)*	SLAM family member 7 (SLAMF7)*
Tumor necrosis factor receptor superfamily member 10A (TNFRSF10A)*	Interleukin-27 (IL-27)*
Leptin (LEP)*	Cathepsin L1 (CTSL1)*
Tumor necrosis factor receptor superfamily member 11A (TNFRSF11A)*	Angiopoietin-1 (ANGPT1)*
Protein AMBP (AMBP)*	Spondin-2 (SPON2)*
Macrophage receptor MARCO (MARCO)*	SLAM family member 5 (CD84)*
Pro-adrenomedullin (ADM)*	C-C motif chemokine 17 (CCL17)*
CD40 ligand (CD40-L)*	C-X-C motif chemokine 1 (CXCL1)*
A disintegrin and metalloproteinase with thrombospondin motifs 13 (ADAM-TS13)*	Receptor for advanced glycosylation and products (RAGE)*
Bone morphogenetic protein 6 (BMP-6)*	Programmed cell death 1 ligand 2 (PD-L2)*
Osteoclast-associated immunoglobulin-like receptor (hOSCAR)*	Pentraxin-related protein PTX3 (PTX3)*
Pro-interleukin-16 (IL16)*	TNF-related apoptosis-inducing ligand receptor 2 (TRAIL-R2)*
**Biomarkers in Chemotaxis (17 proteins)**	
Galectin-3 (Gal-3)†	Integrin beta-2 (ITGB2)†
C-C motif chemokine 24 (CCL24)†	CD166 antigen (ALCAM)†
Azurocidin (AZU1)†	C-C motif chemokine 16 (CCL16)†
Retinoic acid receptor responder protein 2 (RARRES2)†	Plasminogen activator inhibitor 1(PAI)†
Urokinase plasminogen activator surface receptor (U-PAR)†	Monocyte chemotactic protein 1 (MCP-1)†
C-C motif chemokine 15 (CCL15)†	Pulmonary surfactant-associated protein D (PSP-D)†
Interleukin-6 receptor subunit alpha (IL-6RA)†	C-X-C motif chemokine 16 (CXCL16)†
Urokinase-type plasminogen activator (uPA)†	Interleukin-17 receptor A (IL-17RA)†
Platelet-derived growth factor subunit A (PDGF subunit A)†	
**Biomarkers in Angiogenesis (28 proteins)**	
Growth/differentiation factor 2 (GDF-2)*	Vascular endothelial growth factor D (VEGFD)*
Angiopoietin-1 receptor (TIE2)*	Heme oxygenase 1 (HO-1)*
Decorin (DCN)*	Serine/theonine-protein kinase 4 (STK4)*
Placenta growth factor (PGF)*	Interleukin-6 (IL6)*
Leptin (LEP)*	Heat shock 27 kDa protein (HSP 27)*
Trombospondin-2 (THBS2)*	Interleukin-18 (IL-18)*
Pro-adrenomedullin (ADM)*	Angiopoietin-1 (ANGPT1)*
Natriuretic peptides B (BNP)*	Tissue factor (TF)*
Perlecan (PLC)^	C-C motif chemokine 24 (CCL24)^
Neurogenic locus notch homolog protein 3 (Notch 3)^	Plasminogen activator inhibitor 1 (PAI)^
Chitinase-3-like protein 1 (CHI3L1)^	Monocyte chemotactic protein 1 (MCP-1)^
Integrin beta-2 (ITGB2)^	Matrix metalloproteinase-2 (MMP-2)^
Urokinase-type plasminogen activator (uPA)^	Aminopeptidase N (AP-N)^
Ephrin type-B receptor 4 (EPHB4)^	Platelet-derived growth factor subunit A (PDGF subunit A)^
**Biomarkers in Response to hypoxia (17 proteins)**	
Vascular endothelial growth factor D (VEGFD)*	Platelet-derived growth factor subunit B (PDGF subunit B)*
Proto-oncogene tyrosine-protein kinase Src (SRC)*	Angiopoietin-1 receptor (TIE2)*
Pro-adrenomedullin (ADM)*	Heme oxygenase 1 (HO-1)*
Placenta growth factor (PGF)*	Leptin (LEP)*

Matrix metalloproteinase-2 (MMP-2)^	Caspase-3 (CASP-3)^
Platelet-derived growth factor subunit A (PDGF subunit A)^	Monocyte chemotactic protein 1 (MCP-1)^
Transferrin receptor protein 1 (TR)^	Urokinase-type plasminogen activator (uPA)^
Myoglobulin (MB)^	Tissue-type plasminogen activator (t-PA)^
Tumor necrosis factor receptor superfamily member 6 (FAS)^	
**Biomarkers in Inflammatory response (59 proteins)**	
Lymfophotactin (XCL1)*	C-X-C motif chemokine 1 (CXCL1)*
Interleukin-1 receptor-like 2 (IL1RL2)*	Interleukin-4 receptor subunit alpha (IL-4RA)*
Bone morphogenetic protein 6 (BMP-6)*	Interleukin-6 (IL6)*
Lipoprotein lipase (LPL)*	Pentraxin-related protein PTX3 (PTX3)*
Angiopoietin-1 receptor (TIE2)*	CD40 ligand (CD40-L)*
Leptin (LEP)*	TNF-related apoptosis-inducing ligand receptor 2 (TRAIL-R2)*
Receptor for advanced glycosylation end products (RAGE)*	Interluekin-18 (IL-18)*
Lectin-like oxidased LDL receptor 1 (LOX-1)*	Proteinase-activated receptor 1 (PAR-1)*
C-C motif chemokine 3 (CCL3)*	Tissue factor (TF)*
Tumor necrosis factor receptor superfamily member 11A (TNFRSF11A)*	Interleukin-27 (IL-27)*
Tumor necrosis factor receptor superfamily member 10A(TNFRSF10A)*	Galectin-9 (Gal-9)*
Interleuki-17D (IL-17D)*	NF-kappa-B essential modulator (NEMO)*
C-C motif chemokine 17 (CCL17)*	Interleukin-1 receptor antagonist protein (IL-1ra)*
Heme oxygenase 1 (HO-1)*	Angiotensin-converting enzyme 2 (ACE2)*
Tyrosine-protein kinase receptor UFO (AXL)^	Monocyte chemotactic protein 1 (MCP-1)^
Interleukin-2 receptor subunit alpha (IL2-RA)^	C-C motif chemokine 16 (CCL16)^
Transferrin recptor protein 1 (TR)^	Integrin beta-2 (ITGB2)^
Osteoprotegrin (OPG)^	Tumor necrosis factor receptor 1 (TNF-R1)^
Tumor necrosis factor receptor superfamily member 14 (TNFRSF14)^	E-selectin (SELE)^
Tartrate-resistant acid phosphatase type 5 (TR-AP)^	Plasminogen activator inhibitor 1 (PAI)^
Tumor necrosis factor receptor superfamily member 10C (TNFRSF10C)^	C-C motif chemokine 15 (CCL15)^
Scavenger receptor cysteine-rich type 1 protein M130 (CD163)^	Fatty acid-binding protein, adipocyte (FABP4)^
Tumor necrosis factor receptor 2 (TNF-R2)^	ST2 protein (ST2)^
Retinoic acid receptor responder protein 2 (RARRES2)^	C-C motif chemokine 24 (CCL24)^
Peptidoglycan recognition protein 1 (PGLYRP1)^	Junctional adhesion molecule A (JAM-A)^
P-selectin (SELP)^	Tumor necrosis factor receptor superfamily member 6 (FAS)^
Azurocidin (AZU1)^	Chitinase-3-like protein 1 (CHI3L1)^
Interleukin-6 receptor subunit alpha (IL-6RA)^	Interleukin-1 receptor type 1 (IL-1RT1)^
Osteopontin (OPN)^	Interleukin-17 receptor A (IL-17RA)^
Lymphotoxin-beta receptor (LTBR)^	
**Biomarkers in Cell adhesion (62 proteins)**	
Receptor for advanced glycosylation end products (RAGE)*	T-cell surface glycoprotein CD4 (CD4)*
Interleukin-6 (IL-6)*	CD40 ligand (CD40-L)*
Galectin-9 (Gal-9)*	Proto-oncogene tyrosine-protein kinase Src (SRC)*
Protein-glutamine gamma-glutamyltransferase 2 (TGM2)*	Angiopoietin-1 receptor (TIE2)*
Interleukin-18 (IL-18)*	Leptin (LEP)*
Lymphotactin (XCL1)*	SLAM family member 7 (SLAMF7)*
Programmed cell death 1 ligand 2 (PD-L2)*	Interleukin-27 (IL-27)*
P-selectin glycopritein ligand 1 (PSGL-1)*	Interleukin-1 receptor antagonist protein (IL-1ra)*
Stem cell factor (SCF)*	A disintegrin and metalloproteinase with trombospondin motifs 13 (ADAM-TS13)*
Heat shock 27 kDa protein (HSP 27)*	Lectin-like oxidaized LDL receptor 1 (LOX-1)*
Interleukin-4 receptor subunit alpha (IL-4RA)*	Interleukin-1 receptor-like 2 (IL1RL2)*
Angiopoietin-1 (ANGPT1)*	Protein AMBP (AMBP)*
Tyrosine-protein kinase Mer (MERTK)*	Spondin-2 (SPON2)*
Brother of CDO (BOC)*	SLAM family member 5 (CD84)*
Thrombospondin-2 (THBS2)*	Trem-like transcript 2 protein (TLT-2)^
Urokinase-type plasminogen activator (uPA)^	Caspase-3 (CASP-3)^
Collagen alhpa-1 (I) chain (COL1A1)^	Integrin beta-2 (ITGB2)^
Von Willebrand factor (vWF)^	Galectin-3 (Gal-3)^
Tyrosine-protein kinase receptor UFO (AXL)^	P-selectin (SELP)^
Cadherin-5 (CDH5)^	Ephrin type-B receptor 4 (EPHB4)^
Interleukin-2 receptor subunit alpha (IL2-RA)^	Epithelial cell adhesion molecule (Ep-CAM)^
Intercellular adhesion molecule 2 (ICAM-2)^	Epidermal growth factor receptor (EGFR)^

E-selectin (SELE)^	Osteopontin (OPN)^
CD166 antigen (ALCAM)^	Tyrosine-protein phosphatase non-receptor type substrate 1 (SHPS-1)^
Complement component C1q receptor (CD93)^	Tumor necrosis factor receptor superfamily member 6 (FAS)^
Monocyte chemotactic protein 1 (MCP-1)^	Plasminogen activator inhibitor 1 (PAI)^
Insulin-like growth factor-binding protein 2 (IGFBP-2)^	Insulin-like growth factor-binding protein 7 (IGFBP-7)^
Platelet endothelial cell adhesion molecule (PECAM-1)^	Pulmonary surfactant-associated protein D (PSP-D)^
Contactin-1 (CNTN1)^	Tartrate-resistant acid phosphatase type 5 (TR-AP)^
Tumor necrosis factor superfamily member 14 (TNFRSF14)^	Spondin-1 (SPON1)^
Galectin-4 (Gal-4)^	Azurocidin (AZU1)^
**Biomarkers in Proteolysis (34 proteins)**	
Proto-oncogene tyrosine-protein kinase Src (SRC)*	Poly (ADP-ribose) polymerase 1 (PARP-1)*
A disintegrin and metalloproteinase with trombospondin motifs 13 (ADAM-TS13)*	Tissue factor (TF)*
Lectin-like oxidase LDL receptor 1 (LOX-1)*	Matrix metalloproteinase-12 (MMP-12)*
Chymotrypsin C (CTRC)*	Tumor necrosis factor receptor superfamily member 10A (TNFRSF10A)*
Renin (REN)*	Interleukin-6 (IL6)*
Proteinase-activated receptor 1 (PAR-1)*	CathepsinL1 (CTSL1)*
TNF-related apoptosis-inducing ligand receptor 2 (TRAIL-R2)*	Matrix metalloproteinase-7 (MMP-7)*
Angiotensin-converting enzyme 2 (ACE2)*	Galectin-9 (Gal-9)*
Cathepsin Z (CTSZ)^	Tumor necrosis factor receptor superfamily member 6 (FAS)^
Tissue-type plasminogen activator (t-PA)^	Carboxypeptidase A1 (CPA1)^
Metalloproteinase inhibitor 4 (TIMP4)^	Proprotein convertase subtilisin/kexin type 9 (PCSK9)^
Urokinase-type plasminogen activator (uPA)^	Bleomycin hydrolase (BLM hydrolase)^
Tumor nexrosis factor receptor 2 (TNF-R2)^	Kallikrein-6 (KLK6)^
Matrix metalloproteinase-3 (MMP-3)^	Plasminogen activator inhibitor 1 (PAI)^
Caspase-3 (CASP-3)^	Cystatin-B (CSTB)^
Urokinase plasminogen activator surface receptor (U-PAR)^	Azurocidin (AZU1)^
Matrix metalloproteinase-9 (MMP-9)^	Matrix metalloproteinase-2 (MMP-2)^
**Biomarkers in Catabolic process (43 proteins)**	
Matrix metalloproteinase-7 (MMP7)*	Alpha-L-iduronidase (IDUA)*
Decorin (DCN)*	Protein AMBP (AMBP)*
Fatty acid-binding protein, intestinal (FABP2)*	Heme oxygenase 1 (HO1)*
2,4-dienoyl-CoA reductase, mitochondrial (DECR1)*	Cathepsin L1 (CTSL1)*
Brother of CDO (BOC)*	Gastrotropin (GT)*
Fibroblast grwoth factor 23 (FGF23)*	Hydroxyacid oxidase 1 (HAOX1)*
Receptor for advanced glycosylation end products (RAGE)*	Leptin (LEP)*
A disintegrin and metalloproteinase with trombospondin motifs 13 (ADAM-TS13)*	Heat shock 27 kDa protein (HSP 27)*
Angiotensin-converting enzyme 2 (ACE2)*	Matrix metalloproteinase-12 (MMP-12)*
Lipoprotein lipase (LPL)*	Prolargin (PRELP)*
Cathepsin D (CTSD)^	Perlecan (PLC)^
Cathepsin Z (CTSZ)^	Low-density lipoprotein receptor (LDL receptor)^
Fatty acid-binding protein, adipocyte (FABP4)^	Caspase-3 (CASP-3)^
Proprotein convertase subtilisin/kexin type 9 (PCSK9)^	Paraoxonase (PON3)^
Peptidoglycan recognition protein 1 (PGLYRP1)^	Myeloblastin (PRTN3)^
Chitotriosidase-1 (CHIT1)^	Metalloproteinase inhibitor 4 (TIMP4)^
Collagen alpha-1 (I) chain (COL1A1)^	Matrix metalloproteinase-2 (MMP-2)^
Aminopeptidase N(AP-N)^	Tumor necrosis factor receptor 2 (TNF-R2)^
Kallikrein-6 (KLK6)^	Matrix metalloproteinase-3 (MMP-3)^
Retinoic acid receptor responder protein 2 (RARRES2)^	Epidermal growth factor receptor (EGFR)^
Complement component C1q receptor (CD93)^	Myeloperoxidase (MPO)^
Matrix metalloproteinase-9 (MMP-9)^	
**Biomarkers in Ras-MAPK pathway (40 proteins)**	
NF-kappa-B essential modulator (NEMO)*	Proheparin-binding EGF-like growth factor (HB-EGF)*
Stem cell factor (SCF)*	Platelet-derived growth factor subunit B (PDGF subunit B)*
Growth/differentiation factor 2 (GDF-2)*	Angiopoietin-1 (ANGPT1)*
C-C motif chemokine 3 (CCL3)*	Angiopoietin-1 receptor (TIE2)*
Proteinase-activated receptor 1 (PAR-1)*	Leptin (LEP)*
Renin (REN)*	Tumor necrosis factor receptor superfamily member 11A (TNFRSF11A)*
Thrombopoietin (THPO)*	Fibroblast growth factor 21 (FGF21)*

Galectin-9 (Gal-9)*	Proto-oncogene tyrosine-protein kinase Src (SRC)*
Growth hormone (GH)*	Lymphotactin (XCL1)*
Fibroblast growth factor 23 (FGF-23)*	C-C motif chemokine 17 (CCL17)*
Interleukin-18 (IL-18)*	Bone morphogenetic protein 6 (BMP-6)*
Interleukin-6 (IL6)*	Protein AMBP (AMBP)*
CD40 ligand (CD40-L)*	Monocyte chemotactic protein 1 (MCP-1)^
Tumor necrosis factor receptor 2 (TNF-R2)^	C-C motif chemokine 24 (CCL24)^
Interleukin-2 receptor subunit alpha (IL2-RA)^	Growth/differentiation factor 15 (GDF-15)^
Epidermal growth factor receptor (EGFR)^	Platelet-derived growth factor subunit A (PDGF subunit A)^
Tumor necrosis factor receptor superfamily member 14 (TNFRSF14)^	Tumor necrosis factor receptor superfamily member 6 (FAS)^
Chitinase-3-like protein 1 (CHI3L1)^	Lymphotoxin-beta receptor (LTBR)^
C-C motif chemokine 16 (CCL16)^	Osteoprotegerin (OPG)^
Interleukin-6 receptor subunit alpha (IL-6RA)^	C-C motif chemokine 15 (CCL15)^

* = Included in CVD II; † = Included in CVD III

Table S2. The genetic variants observed in the patients recruited from Paediatric Heart Centre in Lund, Sweden between 2009-2018. The analytical report was issued by an accredited laboratory according to internationally recognised standards, and the variants was determined pathogenic or likely pathogenic at the time for the analyse.

Status	Gene	position	GnomAD version 4.0	Alleles
HCM	MYBPC3 NM_000256.3	c.3697T>C p.(Gln1233ter)	0.000009579	14
HCM	MYBPC3 NM_000256.3	c.3697T>C p.(Gln1233ter)	0.000009579	14
HCM	MYBPC3 NM_000256.3	c.1803del p.(Thr602Profs)*61	0	0
HCM	MYBPC3*			
HCM	MYBPC3 NM_000256.3	c.3697T>C p.(Gln1233ter)	0.000009579	14
HCM	unknown			
G+P-	MYBPC3 NM_000256.3	c.2429G>A p.(Arg810His)	0.00007188	116
G+P-	TNNT2 NM_001001430.3	c.856 C>T p.(Arg286Cys)	0.0024692	6
G+P-	TNNT2 NM_001001430.3	c.856 C>T p.(Arg286Cys)	0.0024692	6
G+P-	MYBPC3 NM_000256.3	c.2429G>A p.(Arg810His)	0.00007188	116
HCM	unknown			
G+P-	MYBPC3 NM_000256.3	c.1505G>A p.(Arg502Gln)	0	0
HCM	MYH7 NM_000257.3	c.746 G>A p.(arg249Gln)	0	0
HCM	unknown			
G+P-	MYH7 NM_000257.3	c.5135 G>A p.(Arg1712Gln)	0.00001673	27
HCM	TCAP NM_003673.4	c.316C>T p.(Arg106Cys)	0	0
HCM	MYBPC3 NM_000256.3	c..2490dup p.(His831Serfs*2)	0.000003718	6
HCM	MYBPC3 NM_000256.3	c..2490dup p.(His831Serfs*2)	0.000003718	6
HCM	MYBPC3 NM_000256.3	c..2490dup p.(His831Serfs*2)	0.000003718	6
HCM	MYBPC3 NM_000256.3	c..2490dup p.(His831Serfs*2)	0.000003718	6
HCM	MYBPC3 NM_000256.3	c.2320G>A p.(Ala774Thr)	0.00002772	43
G+P-	TNNT2 NM_001001430.3	c.856C>T, p.(Arg286Cys)		
HCM	MYH7 NM_000257.4	c.1207 C>T p.(Arg403Trp)	0	0

HCM	MYH7 NM_000257.4**	c.2155 C>G p.(Arg719Gly)	0	0
G+P-	MYBPC3 NM_000256.3	c.1658del, p.(Asp553Alafs2*)	0	0
G+P-	MYBPC3 NM_000256.3	c.1658del, p.(Asp553Alafs2*)	0	0
HCM	ABCC9 NM_005691.3	c.3275 T>G p.Ile1092Ser	0.00002231	36
G+P-	MYBPC3 NM_000256.3	c.710 A>C p.(Tyr237Ser)	0	0
HCM	MYH7 NM_000257.4	c.1988 G>A p.(Arg663His)	0.000009916	16
G+P-	MYH7 NM_000257.4	c.1988 G>A p.(Arg663His)	0.000009916	16
HCM	MYH7 NM_000257.4	c.1988 G>A p.(Arg663His)	0.000009916	16
HCM	TNNI3***			
HCM	MYBPC3 NM_000256.3	c.2373dupG p.(Trp792Valf41*)	0.00002686	42
HCM	MYBPC3 NM_000256.3	c.2373dupG p.(Trp792ValfS41*)	0.00002686	42
G+P-	MYBPC3 NM_000256.3	c.2429G>A p.(Arg810His)	0	0
G+P-	MYH7 NM_000257.4	c.1063 G>A p.(Ala355Thr9	0	0
G+P-	MYH7 NM_000257.4	c.1063 G>A p.(Ala355Thr)	0	0
G+P-	MYH7 NM_000257.4	c.1063 G>A p.(Ala355Thr)	0	0
HCM	PRKAG2 NM_016203.4	c.1589 A>G p.(His530Arg)	0	0
HCM	MYBPC3 NM_000256.3	c.2373dupG p.(Trp792Valfx41*)	0.00002686	42
HCM	MYBPC3 NM_000256.3	c.2373dupG p.(Trp792Valfs41*)	0.00002686	42
HCM	unknown			
HCM	unknown			
G+P-	MYH7 NM_000257.4	c.427 C>T p. (Arg143Trp)	0.00001239	20
G+P-	MYH7 NM_000257.4	c.427 C>T p. (Arg143Trp)	0.00001239	20
G+P-	MYBPC3 NM_000256.3	c.2670G>A p.(Trp890Ter)	0	0
HCM	MYH7 NM_000257.4	c.5135 G>A p.(Arg1712Gln)	0.00001673	27

* Original answer from 2005, not convertable to updated nomenclature; ** A secondary gene in MYBPC3 reported; *** No original answer available.

Table S3. Clinical markers compared between HCM and age- and sex-matched controls (Matched Controls) and between genotype-positive, phenotype-negative (G+P-) individuals and age- and sex-matched controls (Matched Controls) presented as mean and standard deviation (SD).

**Clinical markers**	**HCM** (n=29)	**Matched Controls** (n=29)	**p-value***	**G+P-** (n=17)	**Matched Controls** (n=17)	**p-value***
**CK-MB** ug/L (<5)	2.9 (2.0)	2.3 (1.8)	0.212	2.7 (1.3)	3.2 (5.5)	0.810
**ASAT** ukat/L (<0.6)	0.5 (0.1)	0.5 (0.3)	0.991	0.5 (0.1)	0.5 (0.4)	0.547
**ALAT** ukat/L (<0.6)	0.3 (0.1)	0.2 (0.1)	0.211	0.2 (0.06)	0.2 (0.08)	0.702

**CRP** Mg/ml (<5)	1.6 (1.7)	1.1 (1.2)	0.247	4.0 (10.7)	2.6 (6.8)	0.650
**Sodium** mmol/L	139.6 (1.9)	139.0 (2.9)	0.368	140.1 (3.3)	139.4 (1.9)	0.437
**Potassium** mmol/L	4.5 (0.9)	4.7 (1.2)	0.686	5.2 (1.4)	4.6 (0.4)	0.160
**Creatinine** umol/L	72.6 (19.5)	75.6 (14.5)	0.515	57.7 (16.9)	67.3 (13.9)	0.066

* Between-group differences were evaluated using Two-Sided t-test; p value <0.05 was considered statistically significant

Table S4. Descriptive statistic and multivariable logistic regression models, adjusted for age and sex in pathophysiological pathways when comparing HCM (n=29) with age- and sex-matched controls (Matched Controls) (n=29) in each model. Significant proteins in each pathway are presented as increased or decreased OR for HCM. Significant proteins are dichotomized above or below the median value of each protein in the control groups and included in a binary logistic regression model for each pathway, adjusted for age and sex.

	**HCM** (n=29)	**Matched controls** (n=29)	**Model with continues protein values** OR (95% CI)	**p-value^**	**Model with dichotomized protein values** OR (95% CI)	**p-value^**
**Significant proteins remaining in Ras MAPK** (40 proteins analysed)*						
**FGF-21**	5.83 (2.17)	3.58 (1.40)	53.09 (1.72-1639)	**0.023**	10.0 (2.5-39.6)	**0.001**
**IL2-RA**	4.46 (0.72)	4.80 (0.49)	0.002 (“0”-0.83)	**0.043**		n.s.
**IL-6RA**	12.87 (0.74)	13.26 (0.51)	0.005 (“0”-0.55)	**0.027**	0.26 (0.07-0.95)	**0.041**
**Significant proteins remaining in Cell adhesion** (62 proteins analysed)*						
**PSGL-1**	6.12 (0.34)	5.80 (0.20)	$4.65*10^{+12}$ ($19.5-1.1*10^{+24}$)	**0.029**	8.6 (1.9-39.0)	**0.005**
**IL-1ra**	4.33 (1.06)	3.88 (0.73)	2722 (2.7-2749708)	**0.025**		n.s.
**ICAM-2**	5.72 (0.77)	5.84 (0.74)	662 (1.1-389510)	**0.046**		n.s.
**IL-6**	3.72 (1.57)	3.73 (0.75)	0.013 (0-0.58)	**0.025**		n.s.
**EGFR**	3.75 (0.50)	3.91 (0.55)	$5.7*10^{-11}$ (“0”-0.03)	**0.022**	0.13 (0.03-0.65)	**0.013**
**Significant proteins remaining in Response to hypoxia** (17 proteins analysed)*						

**ADM**	8.15 (0.70)	7.75 (0.75)	5.1 (1.54-17.0)	**0.008**		n.s
**MMP-2**	4.47 (0.45)	4.69 (0.64)	0.093 (0.016-0.54)	**0.008**		n.s
**Significant proteins remaining in Chemotaxis** (17 proteins analysed)*						
**IL-6RA**	12.87 (0.74)	13.26 (0.51)	0.22 (0.06-0.81)	**0.022**		n.s.
**Significant proteins remaining in Immune response** (38 proteins analysed)*						
**PIgR**	7.13 (0.40)	6.96 (0.26)	33427 (15-71010299)	**0.008**		n.s.
**Gal-9**	9.36 (0.44)	9.02 (0.48)	60.6 (1.12-3275.2)	**0.044**	5.91 (1.8-19.8)	**0.004**
**PD-L2**	3.16 (0.81)	3.23 (0.67)	0.023 (0.001-0.55)	**0.020**		n.s.
**hOSCAR**	10.89 (0.38)	10.92 (0.36)	0.002 ("0"-0.45)	**0.025**		n.s.
**Significant proteins remaining in Angiogenesis** (28 proteins analysed)*						
**DCN**	5.37 (0.51)	5.21 (0.42)	133.5 (1.0-17420)	**0.049**		n.s.
**PAI**	4.64 (2.28)	4.45 (1.33)	40.5 (1.9-873.7)	**0.018**		n.s.
**MMP-2**	4.47 (0.45)	4.69 (0.63)	$6.1*10^{-6}$ ("0" – 0.03)	**0.005**		n.s.
**ITGB2**	6.83 (0.48)	6.74 (0.95)	164.6 (1.8-14871)	**0.026**		n.s.

* NPX = normalized protein expression values, Olink Proteomics' arbitrary unit on log2 scale, presented as median values and inter quartile range; "0" = <0.001; p^ = p value <0.05 was considered statistically significant; n.s.=non-significant.

Table S5. Descriptive statistic and multivariable logistic regression models, adjusted for age and sex, in pathophysiological pathways when comparing phenotype-negative, genotype-positive individuals (G+P-) (n=17) with age- and sex-matched controls (Matched Controls) (n=17) in each model. Significant proteins in each pathway are presented as increased or decreased OR for G+P-. Significant proteins are dichotomized above or below the median value of each protein in the control groups and included in a binary logistic regression model, adjusted for age and sex, for each pathway.

	**G+P-**	**Matched controls**	**Model with continues protein values**		**Model with dichotomized protein values**	
	**(n=17)**	**(n=17)**	OR (95% CI)	**p-value^**	OR (95% CI)	**p-value^**
**Significant proteins remaining in Chemotaxis** (17 proteins analysed)*						
**ALCAM**	6.13 (0.58)	6.32 ((0.96)	0.047 (0.004-0.62)	**0.017**		n.s.

**Significant proteins remaining in Immune response** (38 proteins analysed**)***						
**ADAM-TS13**	6.52 (0.32)	6.22 (0.32)	598 (5.8-61607)	**0.007**	11.2 (1.2-105)	**0.034**
**Significant proteins remaining in Angiogenesis** (28 proteins analysed)*						
**TIE2**	8.66 (0.43)	8.31 (0.85)	200.0 (1.1-37978)	**0.048**	65.5 (3.7-1165)	**0.004**
**AP-N**	5.72 (0.54)	6.02 (0.70)	0.0 ("0"-0.28)	**0.019**	0.08 (0.008-0.81)	**0.032**
**Significant proteins remaining in Proteolysis** (34 proteins analysed)*						
**TIMP4**	3.59 (0.85)	4.15 (0.60)	$1.44*10^{-8}$ ("0"-0.5)	**0.042**	0.056 (0.006-0.54)	**0.013**
**PAI**	3.80 (1.60)	4.58 (1.53)	0.005 ("0"-0.6)	**0.030**	0.084 (0.009-0.80)	**0.031**
**U-PAR**	5.77 (0.41)	6.02 (0.80)	4986370 ($2.86-8.7*10^{+12}$)	**0.035**		n.s.

* NPX=normalized protein expression values, Olink Proteomics' arbitrary unit on log2 scale, presented as median values and inter quartile range; "0" = <0.001; p^ = p value <0.05 was considered statistically significant; n.s.=non-significant.
